# Machine learning prediction models for different stages of non-small cell lung cancer based on tongue and tumor marker: a pilot study

**DOI:** 10.1186/s12911-023-02266-5

**Published:** 2023-09-29

**Authors:** Yulin Shi, Hao Wang, Xinghua Yao, Jun Li, Jiayi Liu, Yuan Chen, Lingshuang Liu, Jiatuo Xu

**Affiliations:** 1 The Office of Academic Affairs, Shanghai, 201203 China; 2 College of Traditional Chinese Medicine, Shanghai University of Traditional Chinese Medicine, Shanghai, 201203 China; 3 Longhua Hospital, Shanghai University of Traditional Chinese Medicine, Shanghai, 200032 China

**Keywords:** Non-small cell lung cancer (NSCLC), Clinical stages, Tongue diagnosis, Tumor marker, Prediction model

## Abstract

**Objective:**

To analyze the tongue feature of NSCLC at different stages, as well as the correlation between tongue feature and tumor marker, and investigate the feasibility of establishing prediction models for NSCLC at different stages based on tongue feature and tumor marker.

**Methods:**

Tongue images were collected from non-advanced NSCLC patients (n = 109) and advanced NSCLC patients (n = 110), analyzed the tongue images to obtain tongue feature, and analyzed the correlation between tongue feature and tumor marker in different stages of NSCLC. On this basis, six classifiers, decision tree, logistic regression, SVM, random forest, naive bayes, and neural network, were used to establish prediction models for different stages of NSCLC based on tongue feature and tumor marker.

**Results:**

There were statistically significant differences in tongue feature between the non-advanced and advanced NSCLC groups. In the advanced NSCLC group, the number of indexes with statistically significant correlations between tongue feature and tumor marker was significantly higher than in the non-advanced NSCLC group, and the correlations were stronger. Support Vector Machine (SVM), decision tree, and logistic regression among the machine learning methods performed poorly in models with different stages of NSCLC. Neural network, random forest and naive bayes had better classification efficiency for the data set of tongue feature and tumor marker and baseline. The models’ classification accuracies were 0.767 ± 0.081, 0.718 ± 0.062, and 0.688 ± 0.070, respectively, and the AUCs were 0.793 ± 0.086, 0.779 ± 0.075, and 0.771 ± 0.072, respectively.

**Conclusions:**

There were statistically significant differences in tongue feature between different stages of NSCLC, with advanced NSCLC tongue feature being more closely correlated with tumor marker. Due to the limited information, single data sources including baseline, tongue feature, and tumor marker cannot be used to identify the different stages of NSCLC in this pilot study. In addition to the logistic regression method, other machine learning methods, based on tumor marker and baseline data sets, can effectively improve the differential diagnosis efficiency of different stages of NSCLC by adding tongue image data, which requires further verification based on large sample studies in the future.

**Supplementary Information:**

The online version contains supplementary material available at 10.1186/s12911-023-02266-5.

## Introduction

The International Agency for Research on Cancer (IARC) released the most recent global cancer data [1] in 2020, revealing that lung cancer is the most common cancer in men, the second most common cancer in women after breast cancer, and the leading cause of cancer death. Non-small cell lung cancer (NSCLC) is the most common histological type of lung cancer, accounting for 80–85% of all lung cancer cases, with high morbidity and mortality [2]. Lung cancer patients have a 5-year survival rate of 10–20%, and its prevention, screening, treatment, and reduction of the economic burden associated with lung cancer treatment have become an urgent problem to be solved [3]. Early detection, diagnosis, and treatment of NSCLC are critical for improving patient prognosis and survival rates. Different clinical stages of NSCLC patients receive different treatment methods, and their prognosis varies. Surgery is an effective treatment option for early lung cancer. Surgery can also be used to reduce the tumor burden in patients with locally advanced lung cancer, in conjunction with postoperative radiotherapy and chemotherapy, and the survival period can be effectively extended [4]. Treatment options for patients in advanced stages are limited due to tumor metastasis. Traditional Chinese Medicine (TCM) has specific characteristics and benefits in the treatment of advanced lung cancer. It can effectively reduce symptoms, stabilize tumors, and improve patients’ quality of life [5]. Therefore, it is of great significance to take effective methods to evaluate the clinical stage of NSCLC patients. At present, clinical staging of NSCLC primarily includes imaging and histological methods, with histological examination serving as the gold standard for NSCLC staging diagnosis. However, this method is invasive, complicated, and costly, causing harm to patients and even leading to tumor proliferation, and its use is limited. Therefore, finding a non-invasive, safe, reliable, and simple staging diagnosis approach for NSCLC is critical.

Tongue diagnosis is an important part of TCM diagnosis, and is one of its distinctive features. Studies have shown that the appearance of the tongue can reflect physiological and pathological changes in the body to some extent, and is closely related to a person’s overall health status. Research shows that there is a correlation between the tongue characteristics of patients with Chronic Kidney Disease (CKD) and the disease itself. By evaluating the tongue image features of CKD patients using an automated tongue diagnosis system, valuable information can be provided to clinical doctors, facilitating early detection and diagnosis of CKD [6]. The color, shape, thickness of the tongue coating, as well as the color of the tongue body, have certain correlations with the development of diabetes. Li Jun et al. have shown that tongue image features can significantly improve the prediction accuracy of diabetes risk models [7]. Tongue diagnosis has clinical potential in predicting the risk and severity of gastroesophageal reflux disease (GERD). It is expected to serve as an initial screening indicator for upper gastrointestinal diseases and assist doctors in non-invasive early diagnosis of GERD [8]. In addition, research indicates that the color value and thickness of the tongue coating during menstruation in patients with primary dysmenorrhea (PD) are significantly lower than those in the control group, the tongue image features obtained by computerized tongue image analysis system can serve as an auxiliary method for syndrome differentiation, evaluating therapeutic effects, and predicting prognosis in PD [9]. With the advancement of TCM diagnostic information technology in recent years, the modernization of TCM has ushered in new opportunities and challenges. In clinical practice, a variety of tongue diagnostic instruments are widely used, and the objective data acquisition and analysis technology based on standardized tongue diagnosis has gradually matured. The key technologies of tongue diagnosis include tongue body and tongue coating separation techniques, as well as feature extraction techniques. In modern tongue diagnosis research, digital image processing technology is widely used to extract features of color and texture, and various machine learning methods are used for analysis, all of which have achieved good results [10–13]. Wang X et al. [14] established a diagnostic model of tooth mark tongue based on a deep convolutional neural network, and the model has good validity and generalization, providing an objective and convenient computer-assisted tongue diagnosis method for tracking disease progression and evaluating efficacy from the perspective of informatics. Xu Q et al. [15] segmented tongue image based on deep neural network and established a multi-task joint learning model. Li J et al. [7] established a diabetes risk warning model based on tongue image by stacking model and ResNet50 model, and the results showed that the model established by combining tongue image data with machine learning had high classification efficiency. Digital tongue diagnosis research has become one of the focus of the modern research of TCM, along with the rapid development of artificial intelligence technology, different machine learning methods, such as logistic regression [16], support vector machine (SVM) [17], neural network [12], and other data mining methods have been widely used in medical research. Quantitative diagnosis of information is carried out through various mathematical models, which has promoted the development of TCM information-based intelligent diagnosis.

Serum tumor marker detection is an examination method for patients which has great clinical value in early diagnosis, efficacy evaluation, and prognosis judgment of lung cancer. Currently, it has been widely used in clinical research and plays an important role in monitoring recurrence and metastasis. The clinical value of carcinoembryonic antigen (CEA), carbohydrate antigen 125 (CA-125), carbohydrate antigen 199 (CA-199), alpha-fetoprotein (AFP), neuron-specific enolase (NSE), cytokeratin 19 fragment (CYFRA21-1), and carbohydrate antigen 15 − 3 (CA15-3) in lung cancer has been widely concerned [18]. Studies have shown that serum ferritin (SF), squamous cell carcinoma-associated antigen (SCC), NSE, CEA, and CYFRA21-1 were highly expressed in NSCLC and have important clinical value in evaluating clinicopathology, the combined detection of these 5 tumor markers can improve the diagnostic value of NSCLC [19]. Zhang H et al. [20] established a prediction model for EGFR mutation in NSCLC based on tumor marker and CT feature, and the model results showed that the prediction model combining tumor marker and CT feature was more accurate than the prediction model using tumor marker or CT feature alone.

Based on this, this pilot study is primarily based on the tongue feature and tumor marker of NSCLC, analyzing the tongue feature of NSCLC in different stages, the correlation between tongue feature and tumor marker, and attempting to establish NSCLC prediction models of different stages based on tongue feature and tumor marker using different machine learning methods, and trying to explore a new, non-invasive, and efficient method for diagnosing NSCLC of different stages, in order to effectively promote the early detection, diagnosis and treatment of NSCLC, as well as improve the survival rate and prognosis of patients with NSCLC. This was an exploratory pilot study, mainly focused on assessing the feasibility of the methodological establishment, emphasizing the accuracy and reliability of data collection, description, and analysis, and providing data and references for subsequent in-depth studies.

## Materials and methods

### Study design and subjects

**Fig. 1:**
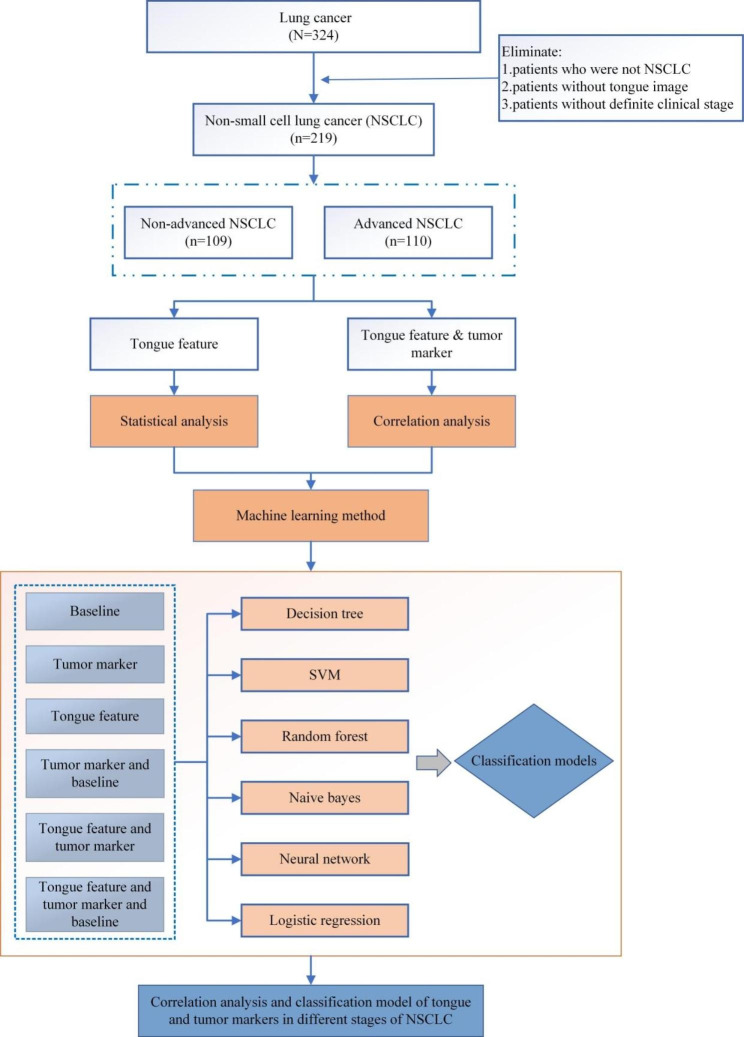
Flow chart

From July 2020 to March 2022, 324 lung cancer patients at Longhua Hospital Affiliated to Shanghai University of Traditional Chinese Medicine’s department of oncology were collected, and their case information, including medical record number, name, gender, medical history information, diagnosis information, and so on, were collected separately. Ethical approval was obtained from the Longhua Hospital affiliated to Shanghai University of Traditional Chinese Medicine Hospital Ethics Committee (registration number 2020LCSY083). Professionally trained graduate students collected standardized tongue image and tumor marker data. A total of 219 NSCLC patients were included in this study, including 109 patients with stages I, II, and III combined into the non-advanced NSCLC group and 110 patients with stage IV in the advanced NSCLC group. All patients were informed and signed informed consent after receiving a clear pathological diagnosis. The research flow chart was shown in Fig. 1.

### Diagnostic criteria

According to the “Clinical Practice Guidelines for Lung Cancer Screening” issued by the National Comprehensive Cancer Network (NCCN) [21] and the fourth edition of the World Health Organization (WHO) “Classification of Lung Tumors” for histological classification of lung cancer [22, 23].

### Inclusion and exclusion criteria

Inclusion criteria: (1) NSCLC diagnosed by pathology or cytology; (2) age ranging from 18 to 90 years; (3) clear pathological staging diagnosis; (4) complete tongue image; and (5) informed and signed informed consent.

Exclusion criteria include: (1) patients who did not meet the inclusion criteria; (2) pregnant or breastfeeding patients; (3) patients with other malignant tumors; (4) patients with systemic acute and chronic infections; and (5) patients with mental illness, unwilling to cooperate, or poor study compliance.

### Collecting clinical data

#### TFDA-1 intelligent tongue diagnosis instrument

**Fig. 2:**
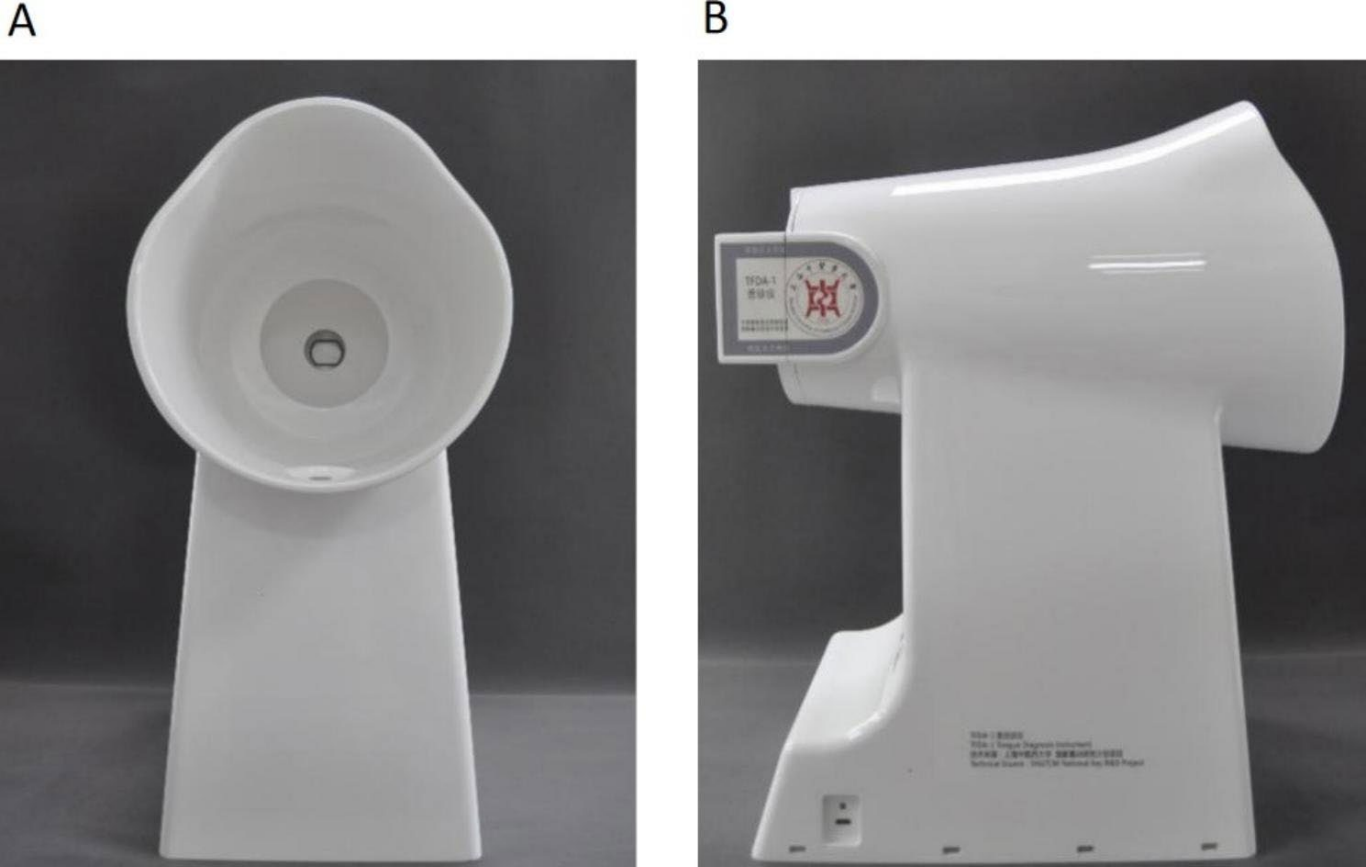
TFDA-1 digital tongue and face diagnosis instrument **A**: front **B**: profile

**Fig. 3:**
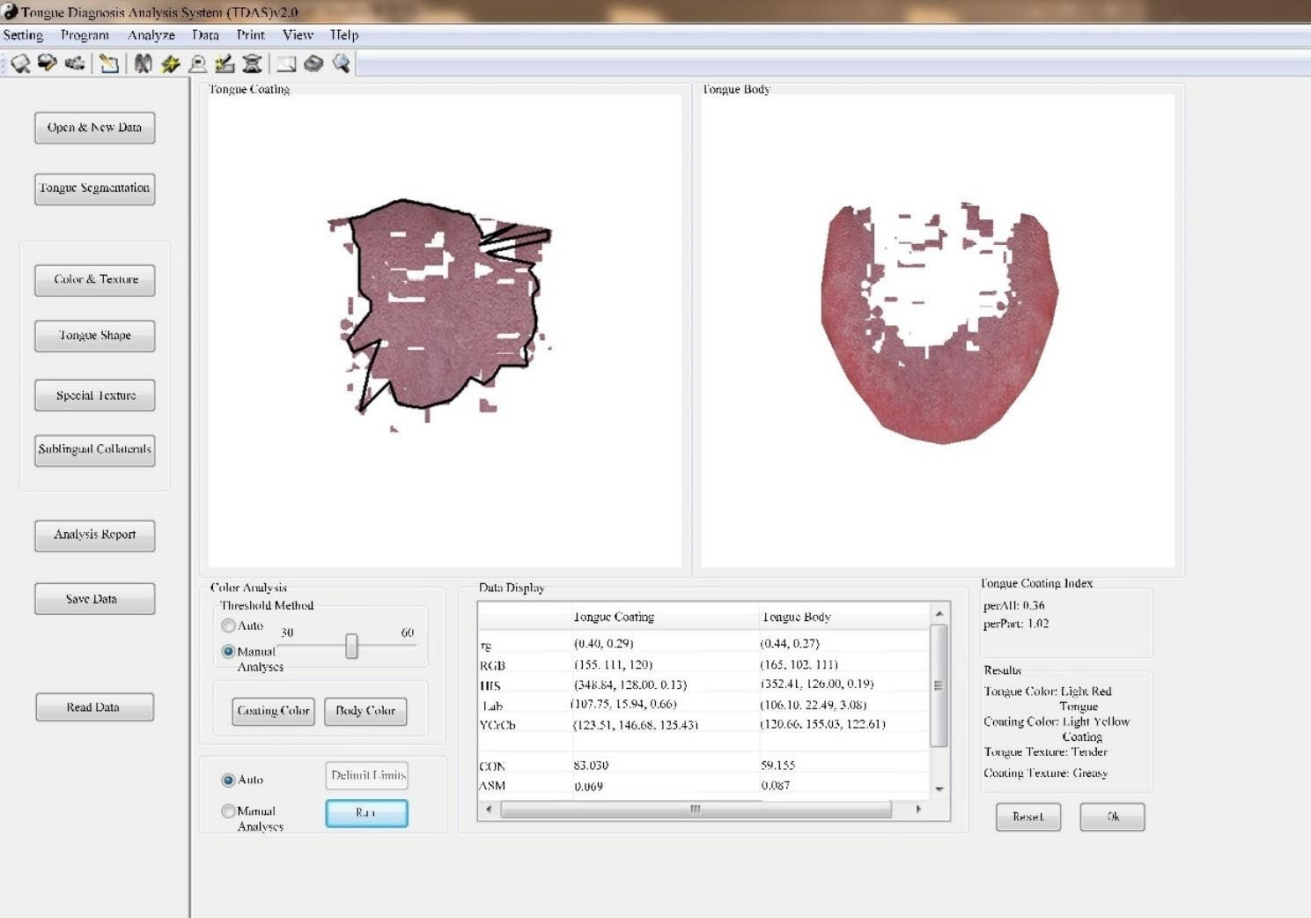
TDAS tongue image analysis system

The Tongue Face Diagnosis Analysis-1(TFDA-1) digital tongue and face diagnosis instrument developed by the project team of the National Key Research and Development Program “TCM Intelligent Tongue Diagnosis System Research and Development” (NO: 2017YFC17033301) was used to collect the tongue images of patients, and the tongue image analysis system TDAS was used to analyze the tongue images to obtain the objective tongue features. The TFDA-1 digital tongue diagnosis instrument was shown in Fig. 2 (A) and Fig. 2 (B), and the corresponding tongue image analysis system TDAS was shown in Fig. 3.

All tongue images were collected by researchers with standardized training to ensure the standardization and accuracy of collection. Specific tongue image collection methods were as follows: (1) set the shooting parameters and sterilize the instrument with alcohol; (2) instruct the subjects to place their chin on the mandibular rest of the digital tongue and face diagnosis instrument, relax naturally, open their mouth and stretch out the tongue, let the tongue body relax, tongue surface is flat, the tip of the tongue is downward, and touch the center of the tongue image in the camera to complete the acquisition. (3) examine the photographed tongue image, ensuring that the tongue body is complete and not nervous and that there is no fogging, light leakage, overexposure, or underexposure, and those who do not meet the requirements must be re-shot.

#### Introduction to features of tongue diagnosis

The tongue color index is derived from four different color spaces: RGB, HSI, Lab, and YCrCb. R(Red), G(Green), and B(Blue) represent the three primary colors of red, green, and blue, with values ranging from 0 to 255. “H” stands for Hue, and its angle range is [0, 2π], which means that the angle of red is 0, the angle of green is 2π/3, the angle of blue is 4π/3, and “S” stands for saturation. “I” stands for intensity; “L” stands for lightness, and its value ranges from 0 to 100, representing pure black to pure white, “a” stands for the green-red axis, its value range is [127, -128], “b” stands for the blue-yellow axis, its value range is [127, -128]; “Y” stands for the luminance, which ranges from 16 to 235, and “Cr” and “Cb” denote chrominance, where Cr denotes the difference between the red part of the RGB input signal and the brightness value of the RGB signal, that is, the degree of offset of the current color to red. and Cb represents the difference between the blue part of the RGB input signal and the brightness value of the RGB signal, that is, the degree of offset of the current color to blue; Cr and Cb have a value range of 16 to 240. CON (Contrast), ASM (Angular Second Moment), ENT (Entropy), and MEAN are the tongue texture indexes; perAll and perPart are the tongue coating indexes, where perAll is the ratio of the tongue coating area to the total tongue area and perPart is the ratio of the coating area to the uncoated tongue area. The prefix “TB-“ refers to the tongue body, and “TC-“ refers to tongue coating in this study. In order to better reflect the continuity of data and find the data regularity and real differences, this study rotated TB-H and TC-H by 180° and redefined the H value after rotation.

The tongue features were extracted automatically by computer batch processing, which had good stability. Data preprocessing in this paper was mainly for data outliers. This study we used the box-graph method to determine outliers, in which the interquartile range (IQR) was the difference between the third (upper) and first (lower) quartile (IQR = Q3-Q1). The upper and lower boundary line was also called outlier cutoff point, the upper outlier cutoff point was the upper quartile + 1.5IQR, the lower outlier cutoff point was the lower quartile − 1.5IQR.

In addition, the tumor markers of patients were obtained from the Hospital Information System (HIS), and the specific indexes included CA50, CA242, AFP, NSE, CA72-4, CYFRA21-1, SCC, CEA, CA125, CA15-3, and CA19-9.

### Statistical analysis

SPSS 25.0 was used for statistical analysis, count data were expressed as percentage N (%), Pearson χ^2^/Fisher’s exact test was used for comparison between groups, measurement data that followed normal distribution were expressed as “X ± SD”, and those that did not conform were expressed as “Median ( P25, P75)”, T-test analysis was performed for groups followed to normality and homogeneity of variance, and independent sample Kruskal-Wallis U test was performed for those not conforming, and correlation heat maps were performed by GraphPad Prism 8.0. All test results were two-tailed, P < 0.05 was considered statistically significant.

### Modeling with machine learning methods

In this experiment, six machine learning classification algorithms were used to establish differential diagnosis models for different stages of NSCLC, namely decision tree, support vector machines(SVM), random forest, neural network, naive bayes and logistic regression. Classification models were built using six data sets: “baseline”, “tumor marker”, “tongue feature”, “tongue feature and tumor marker,“ and “tongue feature and tumor marker and baseline” from patients with different clinical stages of NSCLC, and make two-class predictions respectively, baseline data here mainly included age and sex. All machine learning processes were done on R package. In addition to random forest, all other machine learning methods have been processed with data scaled. The data were normalized using the method of Z-score. The preprocessing-data method of Z-score is described as the following Eq. (6).

6 $$Z=\frac{X-\mu }{\sigma }$$

Where X denotes an element in a data vector, µ for mean value, and $\sigma$ for standard deviation.

This study we used ten-fold cross-validation to screen and confirm the best parameters for the model. The optimal parameters for each model can be found in Supplementary material 1. After confirming the optimal parameters, the parameters were locked, and we resampled 10 times, with each resampled testing set occupying 30% of the total sample and the training set occupying 70%, to ensure that the evaluation results were not accidental. Then, the 10 evaluation results were averaged to reduce errors caused by unreasonable selection in the test set. The modeling was repeated 10 times for each data set, and the “Mean (Standard Deviations)” of the 10 classification results was used to describe the model’s classification performance.

As evaluation indexes, Accuracy, Precision, F1-score, Sensitivity, and Specificity were used. AUC was the area under the ROC curve, with values ranging from 0.5 to 1, the higher the value, the better the classification effect. Sensitivity, also known as true positive rate, assesses the sensitivity of diagnostic methods to diseases, the greater the sensitivity, the lower the likelihood of a missed diagnosis. Specificity is also known as the true negative rate, the higher the specificity, the greater the likelihood of a correct diagnosis. Accuracy indicates the proportion of the number of correctly classified test instances to the total number of test instances. Precision is the ratio of the number of positive cases correctly classified to the number of positive cases classified. F1-score is a harmonic average based on Recall and Precision, which is to evaluate the Recall and Precision comprehensively. The evaluation indexes were shown in the following formulas:

1 $$Accuracy=\frac{TP+TN}{TP+TN+FP+FN}\times 100\text{\%}$$

2 $$Precision=\frac{TP}{TP+FP}\times 100\text{\%}$$

3 $$Sensitivity=\frac{TP}{TP+FN}\times 100\text{\%}$$

4 $$Specificity=\frac{TN}{TN+FP}\times 100\text{\%}$$

5 $$F1=\frac{2\times Precision\times Sensitivity}{Precision+Sensitivity}$$

TP (True Positive) refers to a positive sample predicted as positive by the model. TN (True Negative) refers to a negative sample predicted by the model to be negative. FP (False Positive) refers to a negative sample predicted to be positive by the model; FN (False Negative) refers to a positive sample predicted to be negative by the model.

## Results

### Baseline data

The baseline data of NSCLC of the two groups with different stages were shown in Table 1.

**Table 1:** Baseline data table

Baseline data		Non-Advanced NSCLC(n = 109)	Advanced NSCLC (n = 110)	Z/χ^2^	*P*
Sex(n/%)	male	67(61.47)	50(45.45)	5.642	0.012
	female	42(38.53)	60(54.55)		
Age		65.00 (58.50,69.00)	67.00 (59.75,74.00)	-2.111	0.035

The gender distribution of the two groups was more male than female in the non-advanced NSCLC group and more female than male in the advanced NSCLC group, and the gender difference between the two groups was statistically significant. In addition, the advanced NSCLC group was older than the non-advanced NSCLC group, and the age difference between the two groups was statistically significant.

### Statistical analysis of tongue feature

The statistical analysis results of tongue features in different clinical stages of NSCLC were shown in Table 2.

**Table 2:** Statistical analysis of tongue features [Mean (SD), Median (P25, P75)]

Domain	Index		Non-Advanced NSCLC (n = 109)	Advanced NSCLC (n = 110)	Z value	*P* value
TB	RGB	R	160.00(156.00,164.00)	159.00(152.00,164.25)	-0.916	0.36
		G	91.07 ± 7.23	109.79 ± 7.38	-1.625	0.104
		B	91.00(86.50,97.00)	91.00(83.00,97.00)**	-2.74	0.006
	HIS	H	179.27(176.75,182.11)	178.44(176.58,180.00)*	-2.226	0.026
		I	114.00(110.00,118.00)	112.00(105.00,119.00)	-1.855	0.064
		S	0.21(0.20,0.24)	0.22(0.20,0.24)	-1.019	0.308
	Lab	L	46.64 ± 2.47	45.66 ± 3.31*	-2.27	0.023
		a	28.16(26.43,30.09)	29.28(27.24,31.22)**	-2.753	0.006
		b	12.09(10.18,13.87)	11.50(9.78,13.24)	-1.242	0.214
	YCrCb	Y	111.96 ± 5.57	87.86 ± 9.16*	-2.256	0.024
		Cr	158.31(156.44,159.85)	158.60(156.88,160.58)	-1.505	0.132
		Cb	118.07(116.53,119.60)	118.58(117.15,119.96)	-1.607	0.108
	Texture index	CON	95.45(81.58,111.56)	91.72(76.86,109.54)	-0.808	0.419
		ASM	0.06(0.06,0.07)	0.07(0.06,0.07)	-1.145	0.252
		ENT	1.28(1.24,1.31)	1.27(1.23,1.31)	-0.921	0.357
		MEAN	0.03(0.03,0.03)	0.03(0.03,0.03)	-0.996	0.319
TC	RGB	R	143.00(122.00,153.50)	134.50(117.00,152.00)	-1.675	0.094
		G	93.00(75.00,103.00)	86.00(68.75,102.00)	-1.514	0.13
		B	91.00(70.50,102.00)	86.00(69.00,102.25)	-0.875	0.382
	HIS	H	181.09(178.04,184.97)	180.00(176.85,183.60)*	-2.074	0.038
		I	109.00(89.50,118.00)	102.50(84.00,118.25)	-1.312	0.19
		S	0.17(0.15,0.20)	0.18(0.15,0.20)	-0.229	0.819
	Lab	L	44.72(37.20,48.66)	42.08(34.66,48.36)	-1.475	0.14
		a	19.96(18.63,21.06)	19.92(18.69,21.44)	-0.575	0.565
		b	9.25(7.35,11.44)	8.47(6.92,10.05)*	-2.202	0.028
	YCrCb	Y	108.30(92.40,116.72)	102.86(87.07,116.15)	-1.428	0.153
		Cr	149.58(148.20,150.80)	149.18(147.84,150.25)	-1.431	0.152
		Cb	120.00(118.54,121.55)	120.89(119.43,122.06)**	-2.715	0.007
	Texture index	CON	115.65(84.75,154.56)	132.24(99.64,169.24)	-1.745	0.081
		ASM	0.06(0.05,0.08)	0.06(0.05,0.07)	-1.194	0.232
		ENT	1.29(1.19,1.36)	1.31(1.22,1.39)	-1.34	0.18
		MEAN	0.03(0.03,0.04)	0.04(0.03,0.04)	-1.474	0.14
	Area index	perAll	0.13(0.04,0.23)	0.14(0.05,0.25)	-0.373	0.709
		perPart	0.69(0.51,1.02)	0.63(0.49,0.84)	-1.379	0.168

vs. non-advanced NSCLC group, *P < 0.05, vs. non-advanced NSCLC group, **P < 0.01

**Fig. 4:**
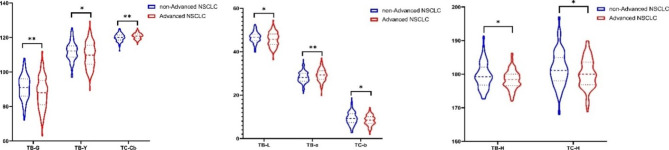
Violin diagram of tongue feature in the non-advanced NSCL and the advanced NSCLC group vs. non-advanced NSCLC group, *P < 0.05, vs. non-advanced NSCLC group, **P < 0.01.

In order to facilitate the observation of the distribution of tongue features with statistically significant differences, GraphPad Prism 8.0 software was used to draw its violin diagram, as shown in Fig. 4.

According to the statistical results, there were statistically significant differences in tongue features between the non-advanced NSCLC and the advanced NSCLC group, and the indexes were TB-B, TB-H, TC-H, TB-L, TB-a, TC-b, TB-Y and TC-Cb, respectively. There was no statistically significant difference in the texture index and tongue coating index between the two groups.

### Correlation analysis of tongue feature and tumor marker

**Fig. 5:**
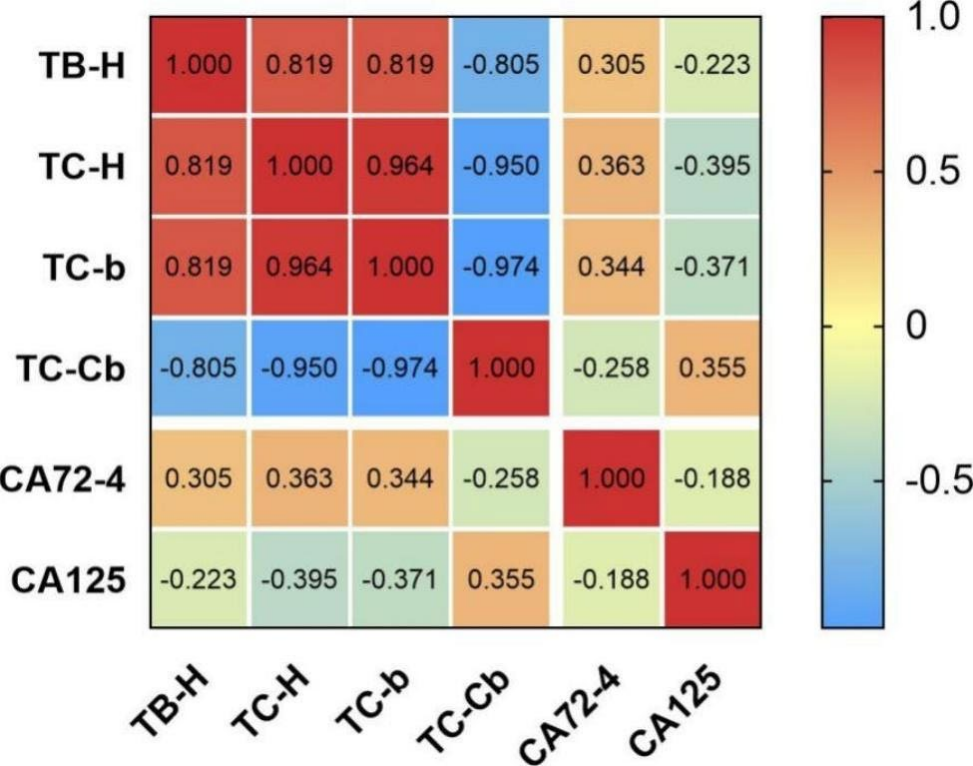
Heat map of correlation between tongue feature and tumor marker in the non-advanced NSCLC group

**Fig. 6:**
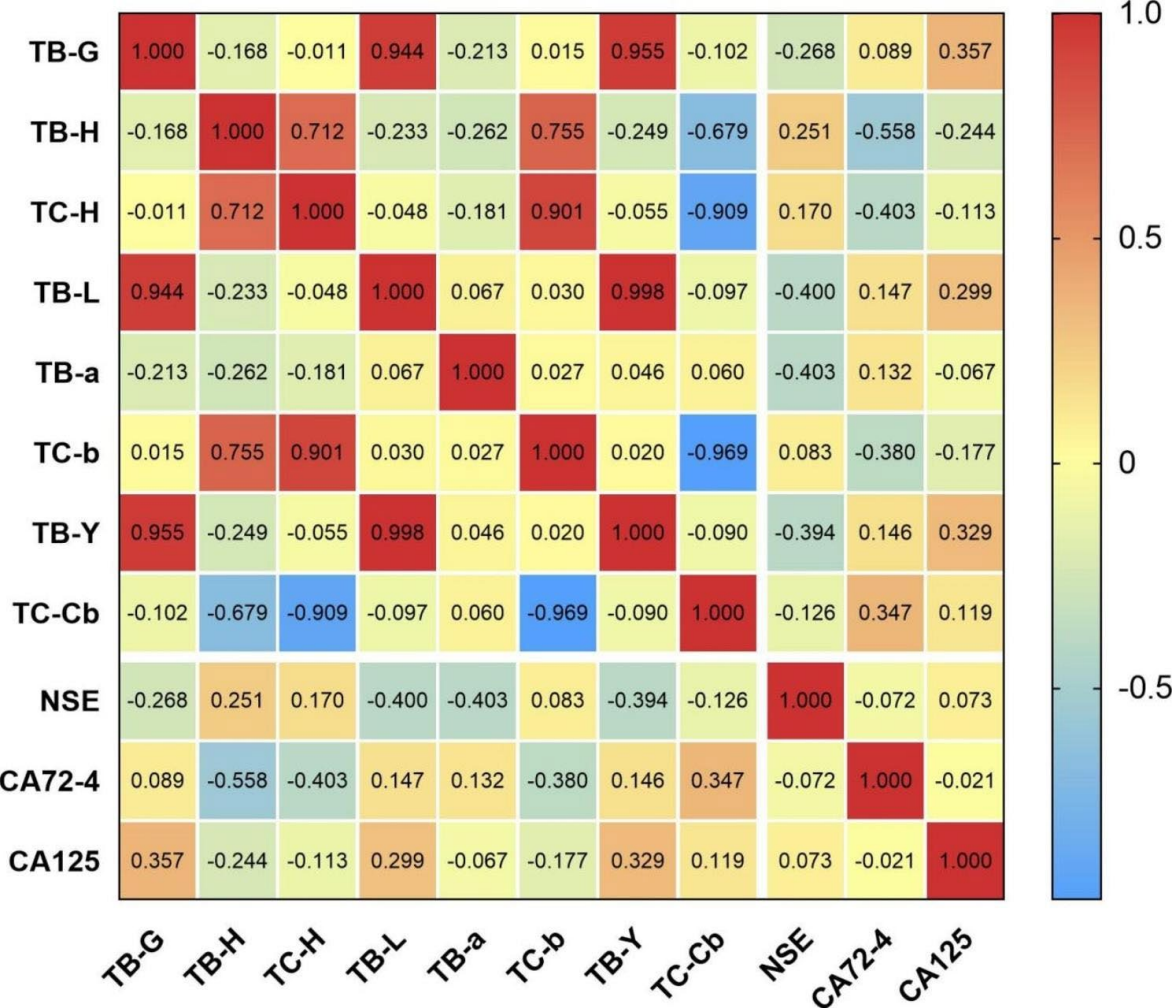
Heat map of correlation between tongue feature and tumor marker in the advanced NSCLC group

In order to further understand the correlation between the index of TCM and Western medicine in patients with different stages of NSCLC, and whether there was any difference in the correlation between the indexes of TCM and Western medicine in patients with different stages, the study analyzed the correlation between tongue feature and tumor marker in the non-advanced NSCLC and the advanced NSCLC group. A total of 107 patients (66 in the non-advanced NSCLC and 41 in the advanced NSCLC group) had complete tongue feature and tumor marker. The indexes of correlation coefficient ≥ 0.3 were used to make correlation heat maps, the statistical results and the correlation heat map of the non-advanced NSCLC group were shown in Table 3; Fig. 5, respectively. Statistical results and the correlated heat map of the advanced NSCLC group were shown in Table 4; Fig. 6 respectively.

**Table 3:** Correlation analysis between tongue feature and tumor marker in the non-advanced NSCLC group

	TB-H	TC-H	TC-b	TC-Cb	CA72-4	CA125
TB-H	1.000					
TC-H	0.819**	1.000				
TC-b	0.819**	0.964**	1.000			
TC-Cb	-0.805**	-0.950**	-0.974**	1.000		
CA72-4	0.305*	0.363**	0.344**	-0.258*	1.000	
CA125	-0.223**	-0.395**	-0.371**	0.355**	-0.188	1.000

*P < 0.05, **P < 0.01

**Table 4:** Correlation analysis between tongue feature and tumor marker in the advanced NSCLC group

	TB-G	TB-H	TC-H	TB-L	TB-a	TC-b	TB-Y	TC-Cb	NSE	CA72-4	CA125
TB-G	1.000										
TB-H	-0.168	1.000									
TC-H	-0.011	0.712**	1.000								
TB-L	0.944**	-0.233	-0.048	1.000							
TB-a	-0.213	-0.262	-0.181	0.067	1.000						
TC-b	0.015	0.755**	0.901**	0.030	0.027	1.000					
TB-Y	0.955**	-0.249	-0.055	0.998**	0.047	0.020	1.000				
TC-Cb	-0.103	-0.679**	-0.909**	-0.097	0.061	-0.969**	-0.090	1.000			
NSE	-0.268	0.251	0.170	-0.400**	-0.403**	0.083	-0.394*	-0.126	1.000		
CA72-4	0.089	-0.558**	-0.403**	0.147	0.132	-0.38*	0.146	0.347*	-0.073	1.000	
CA125	0.357*	-0.244	-0.113	0.299	-0.067	-0.177	0.329*	0.119	0.074	-0.021	1.000

*P < 0.05, **P < 0.01

The statistical results showed that CA125 in the non-advanced NSCLC group was significantly correlated with TC-H, TC-b, TC-Cb, and the correlation coefficients were − 0.395, -0.371, and 0.355 (*P*<0.01), and CA72-4 was correlated with TB-H, TC-H, TC-b, and the correlation coefficients were 0.305, 0.363, 0.344 (*P* < 0.05, *P* < 0.01). There was a correlation between CA72-4 and TB-H, TC-H, TC-b, and TC-Cb in the advanced NSCLC group, and the correlation coefficients were − 0.558, -0.403, -0.380, and 0.347, respectively (*P*<0.05, *P*<0.01), NSE was correlated with TB-a, TB-L, TB-Y, the correlation coefficients were − 0.403, -0.400, -0.394 (*P*<0.05, *P*<0.01), CA125 was correlated with TB-G, TB-Y, and the correlation coefficients were 0.357 and 0.329 (*P* < 0.05).

### Classification model of NSCLC with different clinical stages

Five machine learning classifiers, logistic regression, SVM, random forest, naive bayes, and neural network were used to establish non-advanced NSCLC and advanced NSCLC classification models based on tongue feature, tumor marker, and baseline data. Each dataset was sampled 10 times for each classifier, and the “Mean (Standard Deviations)” of each evaluation index was taken to evaluate the model performance. The classification results of the models were shown in Table 5.

**Table 5:** Classification results of each model based on different data sets [Mean (Standard Deviations)]

Classifier	Models	Sensitivity	Specificity	f1_score	Precision	Accuracy	AUC
decision tree	Model 1	0.261(0.095)	0.885(0.104)	0.348(0.076)	0.671(0.205)	0.612(0.035)	0.626(0.052)
	Model 2	0.448(0.145)	0.817(0.082)	0.514(0.147)	0.638(0.191)	0.664(0.052)	0.628(0.077)
	Model 3	0.340(0.081)	0.805(0.103)	0.417(0.097)	0.570(0.171)	0.606(0.077)	0.570(0.077)
	Model 4	0.412(0.127)	0.826(0.077)	0.489(0.137)	0.632(0.186)	0.655(0.049)	0.615(0.071)
	Model 5	0.487(0.186)	0.776(0.108)	0.525(0.175)	0.608(0.206)	0.658(0.072)	0.658(0.104)
	Model 6	0.410(0.209)	0.799(0.104)	0.451(0.192)	0.618(0.213)	0.63(0.079)	0.615(0.081)
SVM	Model 1	0.265(0.129)	0.932(0.048)	0.403(0.080)	0.738(0.123)	0.639(0.080)	0.547(0.049)
	Model 2	0.210(0.182)	0.878(0.130)	0.272(0.163)	0.643(0.213)	0.606(0.062)	0.536(0.051)
	Model 3	0.380(0.130)	0.831(0.058)	0.452(0.095)	0.614(0.107)	0.633(0.053)	0.548(0.049)
	Model 4	0.417(0.123)	0.844(0.106)	0.506(0.091)	0.690(0.156)	0.667(0.073)	0.589(0.056)
	Model 5	0.526(0.110)	0.846(0.077)	0.600(0.094)	0.721(0.124)	0.715(0.055)	0.606(0.065)
	Model 6	0.583(0.124)	0.837(0.112)	0.644(0.106)	0.743(0.145)	0.736(0.074)	0.655(0.056)
random forest	Model 1	0.485(0.127)	0.802(0.074)	0.540(0.102)	0.639(0.116)	0.670(0.046)	0.680(0.054)
	Model 2	0.443(0.118)	0.838(0.106)	0.523(0.110)	0.690(0.190)	0.673(0.048)	0.735(0.099)
	Model 3	0.425(0.121)	0.772(0.095)	0.474(0.082)	0.579(0.123)	0.621(0.051)	0.679(0.045)
	Model 4	0.414(0.130)	0.883(0.089)	0.511(0.118)	0.740(0.164)	0.688(0.027)	0.769(0.073)
	Model 5	**0.504(0.152)**	**0.866(0.111)**	**0.587(0.154)**	**0.743(0.218)**	**0.712(0.095)**	**0.780(0.074)**
	Model 6	0.514(0.109)	0.869(0.098)	0.597(0.105)	0.756(0.174)	0.718(0.062)	0.779(0.075)
neural network	Model 1	0.343(0.102)	0.876(0.090)	0.441(0.097)	0.688(0.178)	0.648(0.064)	0.676(0.060)
	Model 2	0.354(0.214)	0.834(0.103)	0.420(0.177)	0.594(0.148)	0.642(0.054)	0.642(0.110)
	Model 3	0.505(0.079)	0.771(0.109)	0.547(0.074)	0.625(0.141)	0.655(0.055)	0.660(0.062)
	Model 4	0.378(0.182)	0.817(0.123)	0.429(0.137)	0.617(0.179)	0.624(0.045)	0.631(0.091)
	Model 5	0.488(0.134)	0.827(0.105)	0.561(0.115)	0.686(0.137)	0.694(0.052)	0.741(0.065)
	Model 6	**0.61(0.108)**	**0.874(0.104)**	**0.686(0.095)**	**0.801(0.130)**	**0.767(0.081)**	**0.793(0.086)**
naive bayes	Model 1	0.302(0.078)	0.867(0.092)	0.396(0.056)	0.663(0.191)	0.624(0.053)	0.650(0.067)
	Model 2	0.279(0.134)	0.868(0.101)	0.371(0.132)	0.634(0.172)	0.624(0.087)	0.613(0.096)
	Model 3	0.480(0.119)	0.858(0.068)	0.562(0.087)	0.718(0.096)	0.700(0.042)	0.767(0.064)
	Model 4	0.279(0.134)	0.868(0.101)	0.371(0.132)	0.634(0.172)	0.624(0.087)	0.647(0.093)
	Model 5	0.385(0.101)	0.907(0.065)	0.503(0.091)	0.770(0.096)	0.688(0.070)	0.761(0.078)
	Model 6	0.385(0.101)	0.907(0.065)	0.503(0.091)	0.77(0.096)	0.688(0.070)	0.771(0.072)
logistic regression	Model 1	0.351(0.098)	0.838(0.104)	0.433(0.079)	0.630(0.167)	0.627(0.054)	0.667(0.065)
	Model 2	0.391(0.183)	0.782(0.128)	0.444(0.155)	0.570(0.149)	0.627(0.054)	0.625(0.130)
	Model 3	0.515(0.185)	0.667(0.129)	0.500(0.130)	0.529(0.106)	0.600(0.070)	0.602(0.100)
	Model 4	0.505(0.154)	0.775(0.128)	0.541(0.115)	0.640(0.137)	0.664(0.053)	0.666(0.105)
	Model 5	0.506(0.131)	0.690(0.150)	0.510(0.125)	0.565(0.180)	0.606(0.082)	0.615(0.083)
	Model 6	0.494(0.122)	0.721(0.129)	0.515(0.124)	0.571(0.187)	0.621(0.075)	0.626(0.095)

Note: Model 1,“baseline”, Model 2,“tumor marker”, Model 3, “tongue feature”, Model 4, “tongue feature and baseline”, Model 5, “tongue feature and tumor marker”, Model 6, “tongue feature and tumor marker and baseline”

**Fig. 7:**
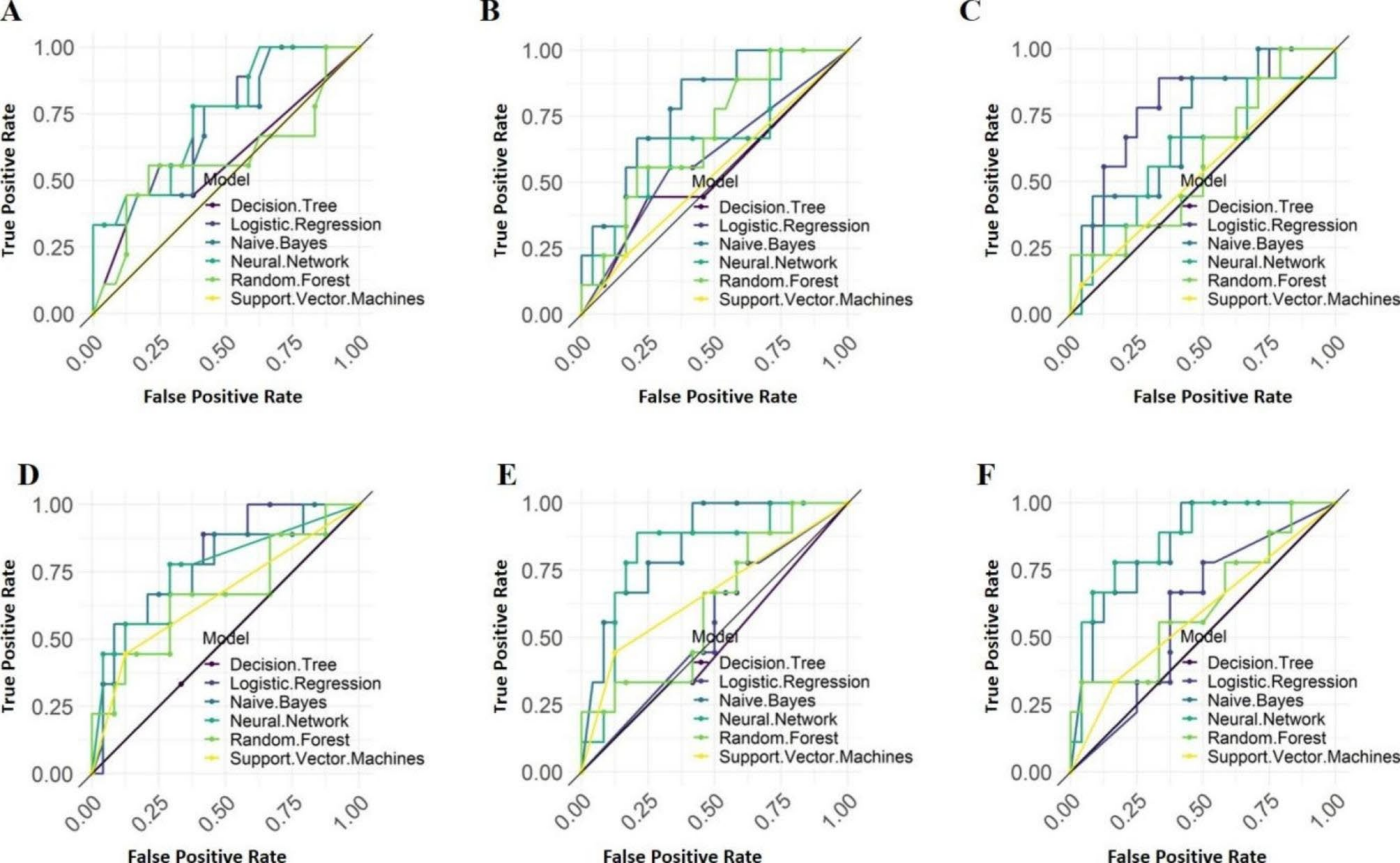
ROC curves of each classifier based on four data sets **A**: Baseline; **B**: Tongue feature; **C**: Tumor marker; **D**: Tumor marker and baseline; **E**: Tongue and Tumor marker; **F**: Tongue and Tumor marker and Baseline

ROC curves of the models based on the six data sets were shown in Fig. 7.

**Fig. 8:**
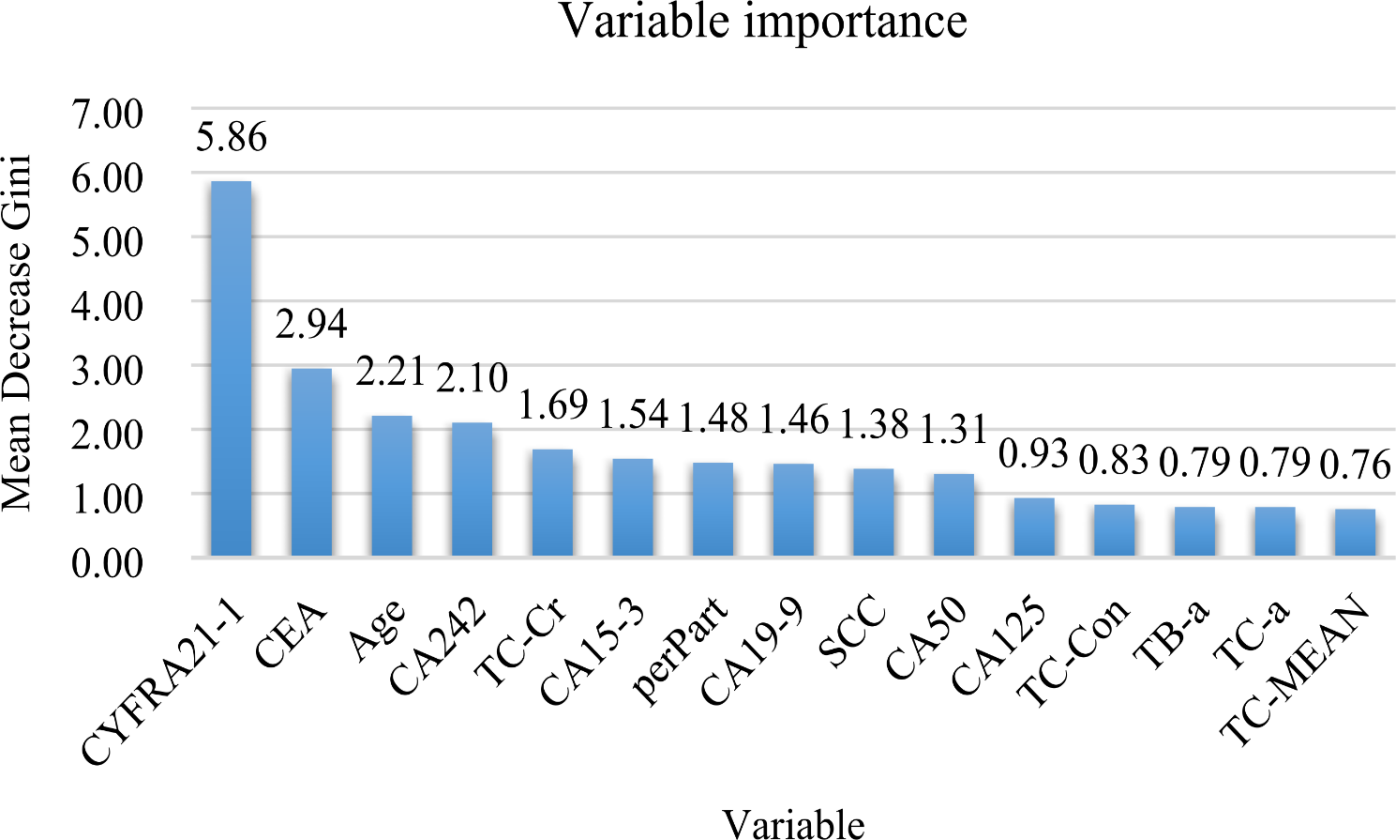
Variable importance based on Random Forest

Gini scores were used to rank the importance of variables. For variables modeled based on random forest method, the importance ranking of the first 15 variables was shown in Fig. 8.

The neural network model based on “tongue feature and tumor marker and baseline” data set had the best classification efficiency, and the confusion matrix of its model was shown in Table 6.

**Table 6:** Confusion matrix of the neural network model

/		Predicted group	
		0	1
Observed group	0	22	2
	1	4	5

Note: “0” represents the non-advanced NSCLC, and “1” represents the advanced NSCLC.

The results showed that different classifiers had different classification effectiveness for different data sets during modeling. Among the machine learning methods tested, SVM, decision tree, and logistic regression performed poorly in models with various stages of NSCLC. In the tumor marker and tongue feature data set, the decision tree performed best, with a model accuracy of 0.658 ± 0.072 and an AUC value of 0.658 ± 0.104. SVM performed best in the baseline and tongue feature and tumor marker data sets, with a model accuracy of 0.736 ± 0.074 and an AUC value of 0.655 ± 0.056. Logistic regression performed best in the baseline data set, with a model accuracy of 0.627 ± 0.054 and an AUC value of 0.667 ± 0.065. Neural network, random forest and naive bayes had better classification efficiency for the data set of tongue feature and tumor marker and baseline. The classification accuracies of the models were 0.767 ± 0.081, 0.718 ± 0.062, and 0.688 ± 0.070, respectively, and the AUCs were 0.793 ± 0.086, 0.779 ± 0.075, and 0.771 ± 0.072.

## Discussion

### Analysis of tongue features in different stages of NSCLC

There were statistically significant differences in tongue features between the non-advanced NSCLC and the advanced NSCLC groups. The indexes mainly focused on the color space index, which was TB-B, TB-H, TC-H, TB-L, TB-a, TC-b, TB-Y, and TC-Cb, respectively. The differences between the two groups were mainly reflected in the intensity and hue of tongue body, the hue of tongue coating, the color of tongue body and tongue coating. The TB-B, TB-H, TB-L, TB-Y, TC-H, and TC-b indexes in the non-advanced NSCLC group were higher than those in the advanced NSCLC group, indicating that the tongue body of the non-advanced NSCLC group was brighter than that of the advanced NSCLC group, and the tongue coating was more yellow. The advanced NSCLC group had higher TB-a and TC-Cr levels than the non-advanced NSCLC group, indicating that the tongue body of the advanced NSCLC group was more reddish purple or cyanotic. The texture index and tongue coating index did not differ statistically between the non-advanced NSCLC and the advanced NSCLC groups, indicating that the tongue texture feature and tongue coating index of different stages of NSCLC could not be distinguished.

### Correlation analysis of tongue feature and tumor marker in different stages of NSCLC

Tongue feature is TCM data, while tumor marker is Western medicine data. Due to the differences in concepts and methods of TCM and Western medicine, the relationship between them has not been systematically established. The essence of the relationship between TCM and Western medicine is the mechanism of disease and syndrome. The correlation analysis of tongue feature and tumor marker in this study will aid in the establishment of a bridge between TCM and Western medicine, allowing for a deeper understanding of the internal mechanism of disease and syndrome, as well as improve the accuracy of disease and syndrome diagnosis. According to the findings of the study, there was a link between TCM and Western medicine in patients with various stages of NSCLC. In the advanced NSCLC group, the number of indexes with statistically significant correlations between tongue feature and tumor marker was significantly higher than in the non-advanced NSCLC group, and the correlations were stronger.

Although some studies have linked CA125, CA15-3, CA19-9, CA72-4, and CYFRA21-1 to lung adenocarcinoma metastasis [24], no studies have confirmed that CA125 can be used as a prognostic marker, and only a small number of studies have discussed its prognostic value in advanced cancer [25], other studies have shown that NSE is an important prognostic factor for advanced locally metastatic NSCLC [18, 26]. CA125 was significantly correlated with TB-H, TC-H, TC-b, and TC-Cb in the non-advanced NSCLC group, CA72-4 was significantly correlated with TB-H, TC-H, and TC-b in the advanced NSCLC group, and CA72-4 was significantly correlated with TB-H, TC-H, TC-b, and TC-Cb in the advanced NSCLC group. CA125 was found to be significantly correlated with TB-G and TB-Y, indicating that CA125 and CA72-4 were related to tongue brightness and yellow tongue coating in both groups of NSCLC patients. The difference was that in the non-advanced NSCLC group, CA125 was correlated with TC-Cb, whereas in the advanced NSCLC group, CA72-4 was correlated with TC-Cb, and TC-Cb was a typical characteristic index of the purple tongue. Furthermore, in the advanced NSCLC group, NSE was significantly correlated with TB-a, TB-L, and TB-Y, indicating that NSE was associated with tongue brightness and redness in patients with advanced NSCLC.

### Analysis of modeling in different stages of NSCLC

The modernization research of diagnostic technology and artificial intelligence technology have greatly promoted the objectification, standardization, and intelligent research of TCM. Through continuous deep learning of big data, machine learning and data mining methods can provide better clinical diagnosis, efficacy evaluation, and prediction models, as well as new methodological support for disease and syndrome research. The organic integration of TCM disease and syndrome research and artificial intelligence technology can effectively promote the development of a TCM intelligent clinical decision-making and efficacy evaluation model with significant practical implications and promising application prospects [27]. This study employed five classifiers, logistic regression, SVM, random forest, naive bayes, and neural network, which were based on tongue feature, tumor marker, tongue feature and tumor marker, tongue feature and tumor marker and baseline data to establish NSCLC classification and diagnosis models of different stages. The results showed that each classifier based solely on tongue feature and solely on tumor marker produced poor classification results, and the model had a high rate of missed diagnosis. All models performed well when combined with tongue feature, tumor marker, and baseline data, implying that single tongue feature and single tumor marker of NSCLC of different stages might not be able to classify or might be affected by the sample size, we should combine multidimensional data and conduct a comprehensive analysis to obtain better classification results when diagnosing NSCLC of different stages. The classifier of neural network based on the tongue feature and tumor marker and baseline data performed best when predict NSCLC at different stages, which suggested that we should give priority to neural network model in differential diagnosis.

## Conclusions

There were statistically significant differences in the tongue feature of NSCLC patients at different clinical stages. In advanced NSCLC patients, there was a stronger correlation between tongue feature and tumor marker. Due to the limited information, single data sources including baseline, tongue feature, and tumor markers cannot be used to identify the different stages of NSCLC. In addition to the logistic regression method, other machine learning methods, based on tumor marker and baseline data sets, can effectively improve the differential diagnosis efficiency of different stages of NSCLC by adding tongue image data. However,  further verification in future studies with large sample is still needed.

### Electronic supplementary material

Below is the link to the electronic supplementary material.

Supplementary Material 1

## Data Availability

The datasets generated and analyzed during the current study are not publicly available due to the confidentiality of the data, which is an important component of the National Key Technology R&D Program of the 13th Five-Year Plan (no. 2017YFC1703301) in China but are available from the corresponding author on reasonable request.

## Abbreviations

IARC: International Agency for Research on Cancer
NSCLC: non-small cell lung cancer
TCM: Traditional Chinese Medicine
SVM: Support vector machine
CEA: Carcinoembryonic antigen
CA125: Carbohydrate antigen 125
CA199: Carbohydrate antigen 199
AFP: Alpha-fetoprotein
NSE: Neuron-specific enolase
CYFRA21-1: Cytokeratin 19 fragment
CA15-3: Carbohydrate antigen 15 − 3
SF: Serum ferritin
SCC: Squamous cell carcinoma-associated antigen
GUI: Graphical User Interface
NCCN: National Comprehensive Cancer Network
TFDA-1: Tongue Face Diagnosis Analysis-1
CON: Contrast
ASM: Angular Second Moment
ENT: Entropy
TB: Tongue Body
TC: Tongue Coating
TP: True Positive
TN: True Negative
FP: False Positive
FN: False Negative
