# Supplementary material for: Machine learning prediction models for different stages of non-small cell lung cancer based on tongue and tumor marker: a pilot study
Source: BMC Med Inform Decis Mak. 2023 Sep 29;23:197. doi: 10.1186/s12911-023-02266-5 (PMC10542664; doi:10.1186/s12911-023-02266-5)
Supplement: Supplementary file 1 — Supplementary Material 1 [file 12911_2023_2266_MOESM1_ESM.docx]

The parameters of each model based on different data sets are as follows：

**Baseline**

Logistic regression = glm(Group~.,family = binomial(link = 'logit'), data = train,)

Decision tree = rpart(Group~.,data = train, cp = 0)

Random forest = randomForest(Group~.,data = train, mtry = 2)

Naive_bayes = naiveBayes(Group~.,data = train, laplace = 0, usekernel = FALSE, adjust = 1)

Neural network = pcaNNet(Group~.,data = train,size = 3, decay = 0)

SVM = svm(Group~.,data = train,cost = 0.25, kernel = 'linear',probability = T)

**Tongue feature**

Logistic regression = glm(Group~.,family = binomial(link = 'logit'), data = train,)

Decision tree = rpart(Group~.,data = train, cp = 0.07317073)

Random forest = randomForest(Group~.,data = train, mtry = 18)

Naive_bayes = naiveBayes(Group~.,data = train, laplace = 0, usekernel = FALSE, adjust = 1)

Neural network = pcaNNet(Group~.,data = train,size = 5, decay = 1e-04)

SVM = svm(Group~.,data = train,cost = 0.25, kernel = 'linear',probability = T)

**Tumor marker**

Logistic regression = glm(Group~.,family = binomial(link = 'logit'), data = train,)

Decision tree = rpart(Group~.,data = train, cp = 0.07317073)

Random forest = randomForest(Group~.,data = train, mtry = 6)

Naive_bayes = naiveBayes(Group~.,data = train, laplace = 0, usekernel = TRUE, adjust = 1)

Neural network = pcaNNet(Group~.,data = train,size = 1, decay = 0.1)

SVM = svm(Group~.,data = train,cost = 0.25, kernel = 'linear',probability = T)

**Baseline and Tumor marker**

Logistic regression = glm(Group~.,family = binomial(link = 'logit'), data = train,)

Decision tree = rpart(Group~.,data = train, cp = 0.07317073)

Random forest = randomForest(Group~.,data = train, mtry = 2)

Naive_bayes = naiveBayes(Group~.,data = train, laplace = 0, usekernel = TRUE, adjust = 1)

Neural network = pcaNNet(Group~.,data = train,size = 5, decay = 0)

SVM = svm(Group~.,data = train,cost = 0.25, kernel = 'linear',probability = T)

**Tongue feature and Tumor marker**

Logistic regression = glm(Group~.,family = binomial(link = 'logit'), data = train,)

Decision tree = rpart(Group~.,data = train, cp = 0.02439024)

Random forest = randomForest(Group~.,data = train, mtry = 23)

Naive_bayes = naiveBayes(Group~.,data = train, laplace = 0, usekernel = FALSE, adjust = 1)

Neural network = pcaNNet(Group~.,data = train,size = 5, decay = 0.1)

SVM = svm(Group~.,data = train,cost = 0.5, kernel = 'linear',probability = T)

**Tongue feature and Tumor marker and baseline**

Logistic regression = glm(Group~.,family = binomial(link = 'logit'), data = train,)

Decision tree = rpart(Group~.,data = train, cp = 0.09756098)

Random forest = randomForest(Group~.,data = train, mtry = 24)

Naive_bayes = naiveBayes(Group~.,data = train, laplace = 0, usekernel = FALSE, adjust = 1)

Neural network = pcaNNet(Group~.,data = train,size = 1, decay = 0.1)

SVM = svm(Group~.,data = train,cost = 1, kernel = 'linear',probability = T)
